# Host Desmin Interacts with RABV Matrix Protein and Facilitates Virus Propagation

**DOI:** 10.3390/v15020434

**Published:** 2023-02-04

**Authors:** Wen Zhang, Yuming Liu, Mengru Li, Jian Zhu, Xiaoning Li, Ting Rong Luo, Jingjing Liang

**Affiliations:** 1 State Key Laboratory for Conservation and Utilization of Subtropical Agro-Bioresources, Guangxi University, Nanning 530004, China; 2 Laboratory of Animal Infectious Diseases, College of Animal Sciences and Veterinary Medicine, Guangxi University, Nanning 530004, China; 3 Department of Pathobiology, School of Veterinary Medicine, University of Pennsylvania, Philadelphia, PA 19104, USA

**Keywords:** Rabies virus, matrix protein, desmin, intermediate filaments, viral-host protein interaction

## Abstract

Microfilaments and microtubules, two crucial structures of cytoskeletal networks, are usurped by various viruses for their entry, egress, and/or intracellular trafficking, including the Rabies virus (RABV). Intermediate filaments (IFs) are the third major component of cytoskeletal filaments; however, little is known about the role of IFs during the RABV infection. Here, we identified the IF protein desmin as a novel host interactor with the RABV matrix protein, and we show that this physical interaction has a functional impact on the virus lifecycle. We found that the overexpression of desmin facilitates the RABV infection by increasing the progeny virus yield, and the suppression of endogenous desmin inhibits virus replication. Furthermore, we used confocal microscopy to observe that the RABV-M co-localizes with desmin in IF bundles in the BHK-21 cells. Lastly, we found that mice challenged with RABV displayed an enhanced expression of desmin in the brains of infected animals. These findings reveal a desmin/RABV-M interaction that positively regulates the virus infection and suggests that the RABV may utilize cellular IFs as tracks for the intracellular transport of viral components and efficient budding.

## 1. Introduction

The Rabies virus (RABV) is a negative-stranded RNA virus of the *Rhabdoviridae* family and is the causative agent of rabies. In the past few decades, efforts to defeat rabies have had substantial success in North America and Western Europe. However, rabies is still a neglected tropical disease in developing countries in Africa and Asia, resulting in a high annual global death count [1,2]. A more comprehensive understanding of the molecular aspects of RABV infection and transmission is warranted for the development of novel and effective post-exposure therapies [3,4].

The RABV encodes five viral proteins, including the nucleoprotein (N), phosphoprotein (P), matrix protein (M), glycoprotein (G), and viral polymerase protein (L), all of which are components of the mature virion. RABV-M is a versatile structural protein that plays multiple roles during virus infection. The central function of the M protein is coordinating virion assembly and budding [5]. To this end, the late (L) budding domain of the M protein hijacks select host proteins (e.g., E3 ubiquitin ligase Nedd4) to facilitate the late step in virus-cell separation [6,7]. The M protein serves as a bridge between the virion envelope and the nucleocapsid core, and also plays a role in viral ribonucleoprotein (RNP) complex condensation, which modulates the balance of viral genome transcription and replication [1,8,9,10]. In addition, the M protein interacts with RelAp43 to modulate the NF-κB pathway and, moreover, cooperates with the P protein to modulate the Jak-Stat pathway, thus contributing to viral immune evasion [11,12,13]. Lastly, the M protein is essential to induce host cell apoptosis and is thought to be one of the determinants of virus pathogenesis [14,15,16]. It should be noted that most of the diverse functions of the M protein are achieved by its interaction and recruitment of specific host factors. Thus, further investigations of the M-host interactome may help to reveal and elucidate the key steps required for RABV infection and transmission.

Towards this end, we utilized a GST pull-down assay coupled with LC-MS/MS analysis to identify the full complement of M-host interactors that may contribute to the virus lifecycle and/or subvert the cellular pathway. Here, we describe the identification of the host intermediate filament protein desmin as a novel interactor with RABV-M. Desmin is one of the members of the type III intermediate filaments (IFs) protein family. As the major structural components of IFs in skeletal, cardiac, and some smooth muscle cells, desmin is important for the maintenance and integrity of the cytoskeleton [17]. Our identification of desmin as a novel host interactor with RABV-M is particularly intriguing since RABV exploits the cytoskeleton network to facilitate different stages of its lifecycle and the development of pathological processes [18,19,20].

In this study, we first demonstrated the association of RABV-M and desmin in vitro, and further confirmed that RABV-M interacts with endogenously-expressed desmin in Baby Hamster Kidney cells (BHK-21). In addition, we found that desmin functions as a positive regulator during the RABV infection; while the overexpression of desmin increased the virus yield, the suppression of endogenous desmin inhibited the virus release. Interestingly, we observed co-localization of RABV-M with the desmin-formed IF bundles in the BHK-21 cells via confocal microscopy. Importantly, our in vivo investigation showed that RABV challenge enhanced the expression level of desmin in a mouse model. In sum, our findings suggest that RABV-M interacts with and hijacks desmin to facilitate the virus infection.

## 2. Materials and Methods

### 2.1. Cells, Plasmids, and Virus

The HEK293T, BHK-21, and BSR cells were maintained in Dulbecco’s Modified Eagle Medium (Corning, NY, USA) supplemented with 10% fetal bovine serum (Gibco, MT, USA), penicillin (100 U/mL)/streptomycin (100 μg/mL) (Invitrogen, MA, USA), and the cells were grown at 37 °C in a humidified 5% CO_2_ incubator. The M genes from RABV strains RC-HL, CVS, and GX01 were cloned into pGEX-4T-1vector for prokaryotic expression. The DNA sequence encoding myc-tagged RABV-M (RC-HL strain) and flag-tagged desmin (Mus musculus) were cloned into the pcDNA 3.0 vector for eukaryotic expression. RABV (RC-HL strain) was grown and titrated, as described previously [21]. For virus titration, the virus in the cell culture supernatant was serially diluted and then incubated with BSR cells that overlay with DMEM, containing 1% methylcellulose and 2% FBS for 48 h. The cells were fixed with methanol and then immunostained with RABV-N antibody (kindly provided by Dr. Minamoto, Gifu University, Japan), and subsequently visualized via Alexa Fluor 488-anti-mouse-IgG secondary antibody (Beyotime, Shanghai, China). The fluorescent focus unit (FFU) assay was used to calculate the virus titer.

### 2.2. GST-Pulldown Assay

The GST alone or GST-tagged RABV-M fusion proteins were expressed in the BL-21 cells and subsequently purified and conjugated into glutathione (GSH) beads (Beyotime, Shanghai, China). The cell extracts from the BHK-21 cells were incubated with the GSH beads described above at 4 °C for 6 h with continuous rotating. The protein complexes were pulled down with beads and subjected to Western blot analysis. The rabbit anti-desmin polyclonal antibody (Proteintech, IL, USA, 16520-1-AP, 1:10,000 dilution) was used to detect desmin, the rabbit anti-RABV-M antiserum (LSBio, WA, USA, LS-C369074, 1:2000 dilution) was used to detect the M fusion proteins, and the mouse anti-GST antibody (Abmart, NJ, USA, M20007, 1:2000 dilution) was used to detect the control GST protein.

### 2.3. Immunoprecipitation Assay

The HEK293T or BHK-21 cells seeded in 6 well plates were transfected with the indicated plasmid combinations using Lipofectamine 2000 reagent (Invitrogen, MA, USA). At 24 h post transfection, the cells were harvested and lysed, and the cell extracts were subjected to Western blot analysis and co-immunoprecipitation. Briefly, the cell extracts were incubated with either mouse IgG or anti-myc (ABclonal, MA, USA, AE010, 1:100 dilution) antibody overnight at 4 °C with continuous rotation, and then the protein A/G agarose beads (Santa Cruz, CA, USA) were added to the mixtures and incubated for 6 h with continuous rotation. After incubation, the beads were collected via centrifugation and washed 5 times. The input cell extracts and immunoprecipitates were then detected by Western blotting with rabbit anti-desmin (Proteintech, IL, USA, 16520-1-AP, 1:10,000 dilution), rabbit anti-RABV-M (LSBio, WA, USA, LS-C369074, 1:2000 dilution) antisera, mouse anti-flag (ABclonal, MA, USA, AE004, 1:2000 dilution), or mouse anti-β-actin (Cwbio, Jiangsu, China, CW0096, 1:2000 dilution) monoclonal antibodies.

### 2.4. Transfection/Infection Assays

The BHK-21 cells seeded in 12 well plates were first transfected with the vector alone or the plasmid encoding desmin (1.0 µg) for 24 h, and subsequently infected with RABV (RC-HL strain) at an MOI of 0.1. Supernatants and cell extracts were harvested at 24, 36, and 48 h post-infection. The released RABV virions were titrated in duplicate via fluorescent focus unit (FFU) assay on the BSR cells. Cellular and viral proteins were detected by Western blotting using the appropriate antibodies. The total RNA from the infected cells was extracted via standard TRIzol (Invitrogen, MA, USA) RNA extraction protocol for quantitative RT-PCR.

### 2.5. siRNA Knockdown

The BHK-21 cells seeded in 12 well plates were first transfected with random siRNA or desmin specific siRNA at a final concentration of 100 nM using Lipofectamine 2000 (Invitrogen, MA, USA). At 24 h post siRNA transfection, the cells were infected with RABV (RC-HL strain) at an MOI of 0.1. The supernatants, cell extracts, and total RNA were harvested at 24, 36, and 48 h post-infection and analyzed by virus titration, Western blotting, and qRT-PCR analyses. The control siRNA sequences include sense-UUCUCCGAACGUGUCACGUTT and antisense-ACGUGACACGUUCGGAGAATT. The desmin siRNA sequences used in this study include siRNA^803–824^, sense-GCAGAAUCGAAUCCCUCAATT, antisense-UUGAGGGAUUCGAUUCUGCTT; siRNA^1369–1417^, sense-GCUCUCAACUUCCGAGAAATT, antisense-UUUCUCGGAAGUUGAGAGCTT.

### 2.6. Quantitative RT-PCR

qRT-PCR was performed using the SYBR Green method (Takara, Japan, RR420). Briefly, 1μg of total RNA served as the template for the first-strand cDNA synthesis in a reaction using an oligo (dT) primer and MMLV reverse transcriptase (Promega, VI, USA, M1701), following the manufacturer’s instructions. The quantitative assessment of RABV N and M mRNAs, and desmin mRNA under standard cycling conditions were performed on LightCycler^®^ 480 System (Roche Diagnostic, IN, USA). The β-actin gene mRNA expression was assessed as a control for all reactions. The primer sequences used in this study includes RABV-N: forward-GGCATTGGCAGATGATGGAACT, reverse-GGCTTGATGATTGGAACTGACTGA; RABV-M: forward-TGATTCCAGGGGCCCTC-TTG, reverse-AAGAGACATGTCAGACCA; Desmin: forward-CCTGGAGCGCAGAATCGAAT, reverse-TGAGTCAAGTCTGAAACCTTGGA.

### 2.7. Indirect Immunofluorescence Assay

The BHK-21 cells expressing RABV-M were washed with cold PBS and fixed with 4% formaldehyde for 15 min at room temperature, then permeabilized with 0.2% Triton X-100 for 10 min. After blocking, the cells were incubated with rabbit anti-desmin (Proteintech, IL, USA, 16520-1-AP, 1:400 dilution) and mouse anti-myc (RABV-M) antibodies for 1 h at room temperature, and then stained with Alexa Fluor 555-anti-Rabbit IgG and Alexa Fluor 488-anti-mouse-IgG secondary antibodies (Beyotime, Shanghai, China). The nuclei were visualized via DAPI staining (Beyotime, Shanghai, China). Microscopy was performed using a Leica SP5 FLIM inverted confocal microscope. Serial optical planes of focus were taken, and the collected images were merged into one by using the Leica microsystems (LAS AF, 5.1.0, Hesse, Germany) software.

### 2.8. In Vivo Studies

All animal experiments were conducted according to the Guidelines for the ethical review of laboratory animal welfare People’s Republic of China (National Standard GB/T 35892-2018) and approved by the Animal Experiment Committee of Guangxi University with the approval number GXU2019-021. The four-week-old female Kunming mice (obtained from Guangxi Medical University, Nanning, China) were intracranially (i.c.) inoculated with DMEM (mock) or 30 μL attenuated RABV RC-HL strain (1000 FFU). At 4 and 7 days post-infection, the mice (two groups of 6) were euthanized, and the brains were collected for total protein and RNA extraction and immunohistochemistry. The total protein and RNA were subjected to Western blotting and qRT-PCR analyses, respectively.

### 2.9. Immunohistochemistry

The brains from the mock and RABV (RC-HL strain) infected mice were collected at 4 and 7 days post infection and fixed in 4% paraformaldehyde, followed by paraffin embedding. The hippocampus was cut into thin sections (5 μm) followed by deparaffinization/rehydration. The slides were submersed into 3% citrate solution and heat-induced epitope retrieval is performed using a pressure cooker for 10 min. The staining steps include washing the slides in dH_2_O for 5 min and incubation in 3% hydrogen peroxide for 20 min, then rewashing the slides in dH_2_O for 5 min and blocking in 5% BSA solution for 30 min. The slides were then incubated with anti-desmin antibody (Proteintech, IL, USA, 16520-1-AP, 1:400 dilution) overnight at 4 °C. The antigen was detected via HRP Polymer and DAB substrate.

### 2.10. Statistical Analysis

Significances were calculated from ≥3 independent experiments in GraphPad Prism (8.0.0, MA, USA) by either the One-way ANOVA-Dunnett’s T3 multiple comparison test or unpaired *t*-test with Welch’s correction, as indicated in the legend.

## 3. Results

### 3.1. Identification of Desmin as a Host Interactor with RABV Matrix Protein

**Figure 1:**
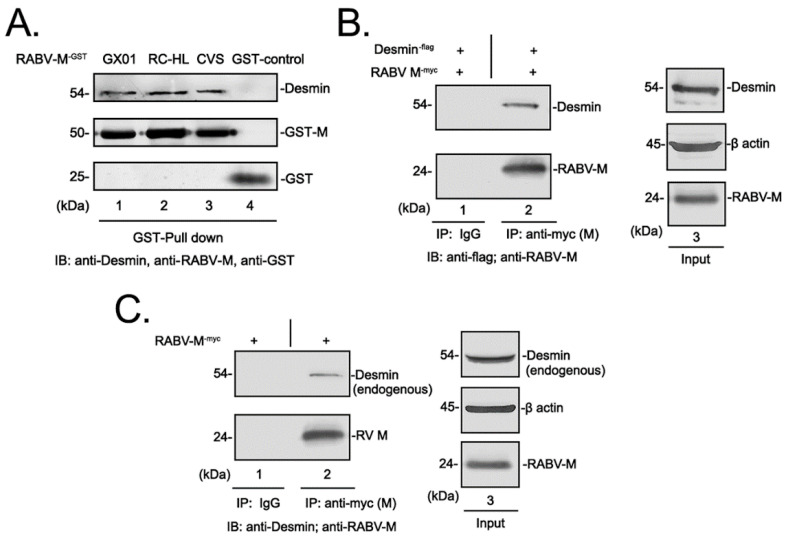
Identification of desmin as a host interactor with RABV matrix protein. (**A**) GST pulldown assays of desmin and M protein from different RABV strains. Purified GST and GST-RABV-M fusion proteins that were incubated with BHK-21 whole cell lysate, desmin, RABV-M, and GST control in pulldown samples were detected by Western blotting (WB) using appropriate antibodies. (**B**) Extracts from HEK293T cells transfected with the indicated plasmids were immunoprecipitated (IP) with either normal mouse IgG or anti-myc antibody, and the precipitated proteins were analyzed by WB using anti-flag (exogenous desmin) or anti-RABV-M antisera. Expression controls for exogenous desmin, RABV-M, and β-actin are shown. (**C**) BHK-21 cells were transfected with plasmid encoding RABV-M alone, cell extracts were IP with either normal mouse IgG or anti-myc antibody, endogenous desmin precipitated with RABV-M were detected by WB using anti-desmin and anti-RABV-M antisera.

To identify host proteins that interact with the RABV matrix protein, we first expressed and purified the GST-tagged RABV-M and constructed a bait by conjugating the GST-M with GSH beads. The baits were subsequently incubated with whole cell lysates from BHK 21 cells to precipitate the RABV-M interacting host proteins, followed by a tandem mass spectrometry (MS) to identify these potential host interactors. We identified desmin in the GST-M samples but not the GST protein control, suggesting that desmin is potential interactor with RABV-M (please see the Data Availability Statement). To verify this finding, a GST-pulldown assay was performed to assess the M-desmin direct interaction in vitro. Briefly, the whole cell lysates from BHK 21 cells were incubated with the purified GST protein alone or the GST-tagged M constructed from the different RABV strains, including the street strain GX01 [22], attenuated strain RC-HL, and standard strain CVS. Western blotting (WB) analysis was utilized to detect desmin, GST-M, and GST proteins in the precipitates. In agreement with our MS analysis, we detected desmin in all of the GST-M precipitates, but not in the GST controls (Figure 1A, compare lanes 1–4), suggesting that desmin interacts with the M protein from various RABV strains.

We next asked whether the RABV-M interacts with desmin in vivo. Briefly, HEK-293T cells were transfected with plasmids encoding myc-tagged M and flag-tagged desmin, and whole cell lysates were incubated with either anti-myc antibody or normal mouse IgG, followed by WB analysis. We detected desmin in the M protein immunoprecipitates, but not in the corresponding IgG control precipitates (Figure 1B, compare lanes 1 and 2), suggesting that the exogenous desmin interacts with the RABV-M in HEK 293T cells. Then, we sought to further verify that RABV-M interacts with endogenously expressed desmin in a biologically relevant cell line. To this end, the BHK-21 cells were transfected with myc-tagged M alone, and the cell lysate was subjected to a co-IP followed by WB analysis. We consistently detected endogenous desmin in the RABV-M immunoprecipitates (Figure 1C). Taken together, these results demonstrated that the intermediate filament protein desmin is a specific host interactor with the RABV matrix protein.

### 3.2. Desmin Facilitates RABV Infection

**Figure 2:**
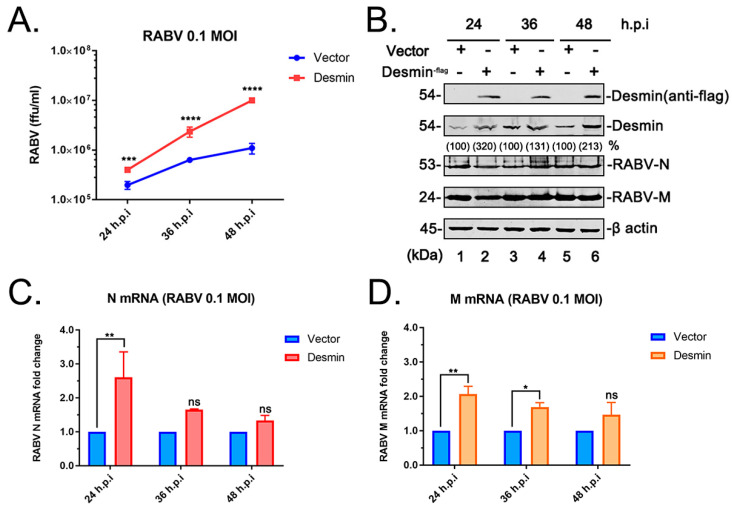
Exogenously expressed desmin facilitates RABV infection. BHK-21 cells were transfected with vector or plasmid encoding flag-tagged desmin, followed by infection with RABV (RC-HL strain) at an MOI of 0.1. (**A**) Virus titration of the supernatants from infected cells at 24, 36, and 48 h post infection (h.p.i). (**B**) Western blotting analysis of desmin and viral proteins RABV-N and M, with β-actin serving as a control, the indicated proteins were quantified using NIH Image-J software (1.53m 28, MD, USA). The infected cell extracts were harvested at 24, 36, and 48 h.p.i. (**C**,**D**) RABV-N (**C**) and RABV-M (**D**) mRNA expression levels were analyzed via qRT-PCR at 24, 36, and 48 h.p.i. Data were normalized to β-actin mRNA expression and are presented as relative fold change vs. controls. Statistical significance of bar graphs was analyzed by unpaired *t*-test with Welch’s correction. ns: not significant, *: *p* < 0.05, **: *p* < 0.01, ***: *p* < 0.001, ****: *p* < 0.0001.

We next sought to investigate whether the physical M-desmin interaction plays a functional role during RABV infection. Towards this end, BHK 21 cells were first transfected with the vector or the plasmid encoding flag-tagged desmin for 24 h and subsequently infected with RABV (RC-HL strain) at an MOI of 0.1. The total RNA, cell lysates, and supernatants from the infected cells were harvested at 24, 36, and 48 h post infection and subjected to qRT-PCR, WB, and virus titration analyses, respectively. We found that the exogenous expression of desmin modestly enhanced the RABV-N (Figure 2C) and RABV-M (Figure 2D) mRNA expression levels at 24 h post infection, but did not alter the viral protein expression levels (Figure 2B). Intriguingly, we observed a significant boost in the yield of progeny virions upon the addition of the exogenous desmin (Figure 2A). Indeed, the virus titration analysis showed that the overexpression of desmin increased virus budding by 10-fold at 48 h post infection, implying that the addition of exogenous desmin facilitates the RABV infection, and that this positive effect is likely due to the enhancement of later stages in the virus lifecycle, such as assembly and budding.

### 3.3. Suppression of Endogenous Desmin Impairs RABV Infection

**Figure 3:**
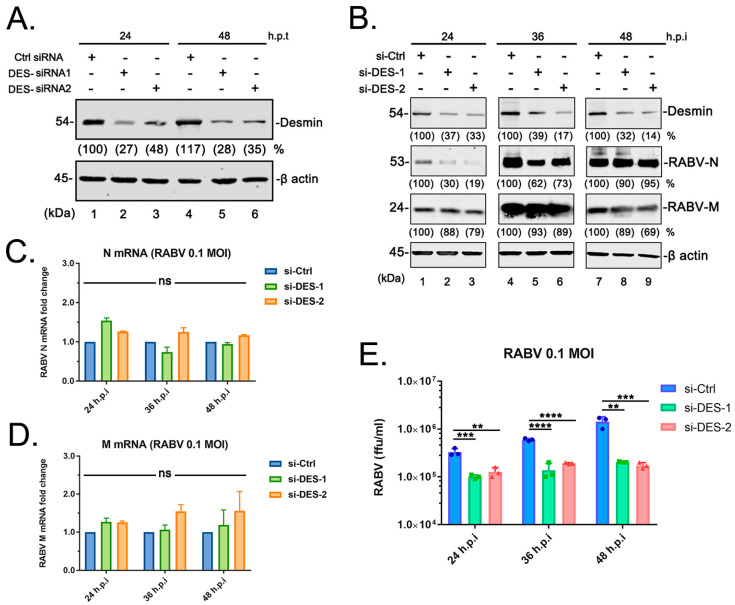
siRNA knockdown of endogenous desmin inhibits RABV infection. (**A**) BHK-21 cells were transfected with control or specific desmin siRNAs, the suppression of endogenous desmin levels was verified via WB and quantified using NIH Image-J software (1.53m 28, MD, USA). (**B**) BHK-21 cells received control or desmin siRNAs were infected with RABV (RC-HL strain) at an MOI of 0.1. The cell extracts were harvested at 24, 36, and 48 h.p.i, followed by WB analysis of desmin and viral proteins RABV-N and M, and β-actin served as a control. The indicated proteins were quantified using NIH Image-J software (1.53m 28, MD, USA). (**C**,**D**) RABV-N (**C**) and RABV-M (**D**) mRNA expression levels in infected cells were analyzed via qRT-PCR at 24, 36, and 48 h.p.i. Data were normalized to β-actin mRNA expression and are presented as relative fold change vs. controls. (**E**) Virus titration of the supernatants from infected cells at 24, 36, and 48 h.p.i. Statistical significance of bar graphs was analyzed by a one-way ANOVA. ns: not significant, **: *p* < 0.01, ***: *p* < 0.001, ****: *p* < 0.0001.

We next sought to determine whether endogenous desmin is required for efficient RABV infection. Toward this end, we first designed and evaluated the siRNAs targeting desmin and selected two combinations of siRNA (Figure 3A). The BHK-21 cells were transfected with the random siRNA control or the desmin-specific siRNAs, followed by the RABV (RC-HL strain) infection at an MOI of 0.1. Again, the total RNA, cell lysates, and supernatants were harvested at 24, 36, and 48 h post infection for qRT-PCR, WB, and virus titration analyses, respectively. We did not detect a significant change in the mRNA levels of RABV-N or M genes (Figure 3C,D) among the cells that received the control or desmin-specific siRNAs, while the suppression of endogenous desmin showed an overall marginal impact on the expression levels of viral proteins (Figure 3B). Notably, we observed a significant decrease in the virus titers in the supernatants from the cells receiving desmin specific siRNAs, compared to those receiving siRNA controls. Interestingly, while the virion yield showed a step-by-step increase along with the infection time from the control cells, the knockdown of the endogenous desmin substantially impaired the virus release (Figure 3E). Taken together, these complementary results confirm that desmin interacts with the RABV matrix protein, and positively regulates the virus infection, likely by supporting the efficient virus release.

### 3.4. RABV Matrix Protein Localizes to the Desmin-Formed Intermediate Filament Bundles

**Figure 4:**
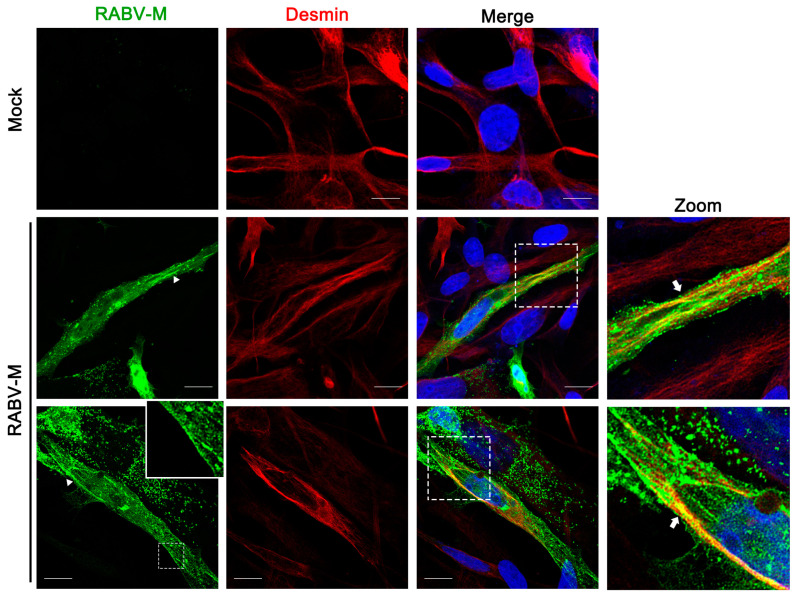
RABV-M co-localizes with desmin in intermediate filament bundles. The RABV-M (green), endogenous desmin (red), and cell nuclei (blue) in BHK-21 cells were visualized via confocal microscopy. The insert panel in the first lane shows the RABV-M localizes to the cell periphery (plasma membrane), while the triangles indicate the bundle-like M protein. The zoom panels highlight the colocalization of RABV-M with desmin in IF bundles (indicate as arrows). Scale bars = 10 µm.

To address the mechanism by which desmin supports RABV egress, we sought to determine whether the RABV-M colocalized with desmin in specific subcellular compartments. For this purpose, we performed an immunofluorescence assay on BHK-21 cells expressing the myc-tagged RABV-M, followed by confocal microscopy to visualize the intracellular patterns of RABV-M and endogenous desmin. It should be noted that desmin is a subunit of intermediate filaments (IFs)—one of the cytoskeletal structural components. As expected, we observed that the endogenous desmin assembles as IF bundles [23] (Figure 4, Desmin), while the RABV-M distributes throughout the cytoplasm (Figure 4, RABV-M), and localizes to the cell periphery (Figure 4, RABV-M, insert panel) [24,25]. Interestingly, we found that a portion of the M protein form bundle-like structures (Figure 4, RABV-M, triangles), and the bundle-like forms of RABV-M overlay with the desmin assembled IFs (Figure 4, Merge). This colocalization of M and desmin in the IF bundles (Figure 4, Zoom, arrows) implies that RABV-M may utilize the IFs during the intracellular transportation, and that desmin may play a role in the M protein-mediated virion assembly and budding.

### 3.5. RABV Infection Enhances Desmin Expression in Mouse Brain

**Figure 5:**
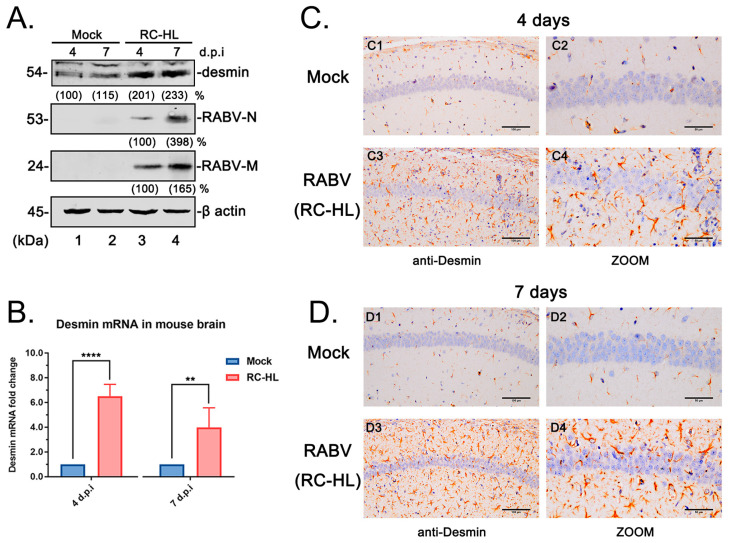
RABV induces desmin expression in the mouse brain. (**A**) Two groups of six 4-week-old Kunming mice were intracranially infected with mock (DMEM) or 1000 FFU RABV (RC-HL strain). The brain tissues from each group were collected at day 4 and 7 post challenge, followed by the WB analysis of desmin and viral proteins N and M, β-actin served as a control. The indicated proteins were quantified using NIH Image-J software (1.53m 28, MD, USA). (**B**) The qRT-PCR analysis of desmin mRNA expression in mouse brain. Data were normalized to β-actin mRNA expression and are presented as relative fold change vs. mock. Statistical significance of bar graphs was analyzed by unpaired *t*-test with Welch’s correction. **: *p* < 0.01, ****: *p* < 0.0001. (**C**,**D**) Immunohistochemistry of desmin in the hippocampus of the mouse brain. The brain samples from mock and RABV-infected mice were collected at day 4 (**C**) and 7 (**D**) post infection. The hippocampus sections were subjected to IHC analysis via staining with the anti-desmin antibody. Scale bars = 100 μm in left panels and 50 μm in zoom panels.

The above data imply that desmin is a novel host interactor with RABV-M that positively regulates the virus infection. We therefore reasoned that RABV may modulate desmin expression to facilitate the virus replication. To address this hypothesis, two groups of six 4-week-old Kunming mice were mock-challenged or challenged with 1000 fluorescent focus-forming units (FFUs, as determined on BSR cells) of RABV (RC-HL strain). Three animals from each group were euthanized on day 4 and day 7 post challenge, and brain samples were collected and assessed by WB, qRT-PCR, and immunohistochemistry (IHC) analyses. Firstly, the tissue proteins were isolated and the levels of RABV-N and M, as well as desmin, were determined via WB (Figure 5A). Both viral proteins were detected at day 4 post challenge and their expression levels increased over time (Figure 5A, lanes 3–4). Interestingly, we found that the levels of desmin were elevated upon the RABV challenge (Figure 5A, compare lanes 1–4). Next, the total RNA from the mouse brain was isolated and desmin mRNA was quantified via qRT-PCR analysis. We observed a significant increase in the levels of desmin mRNA expression (Figure 5B).

Next, four random thin sections from the hippocampus of the mock and RABV infected mouse brains collected at day 4 and 7 post challenge were used for immunohistochemistry (IHC) analysis via immunostaining with anti-desmin antibody. In agreement with the above qRT-PCR and WB analyses, we observed that the RABV infection markedly induced the desmin expression in the mouse hippocampus at both day 4 and 7 post challenge (Figure 5C,D compares the Mock and RABV samples). Moreover, the IHC analysis showed an increase in desmin staining at day 7 compared with that at day 4 in the RABV infected mouse hippocampus (Figure 5, compare panels C3 and D3; C4 and D4), implying that desmin may be further induced during the RABV infection and transmission. In comparison, there was no appreciable change of desmin staining in the mock samples at either day 4 or 7 post challenge (Figure 5, compare panels C1 and D1; C2 and D2). In sum, these data suggest that the expression of desmin in mouse brains is significantly enhanced during RABV infection, further supporting the possible role of desmin as a host interactor that positively regulates the virus replication and transmission in vivo.

## 4. Discussion

**Figure 6:**
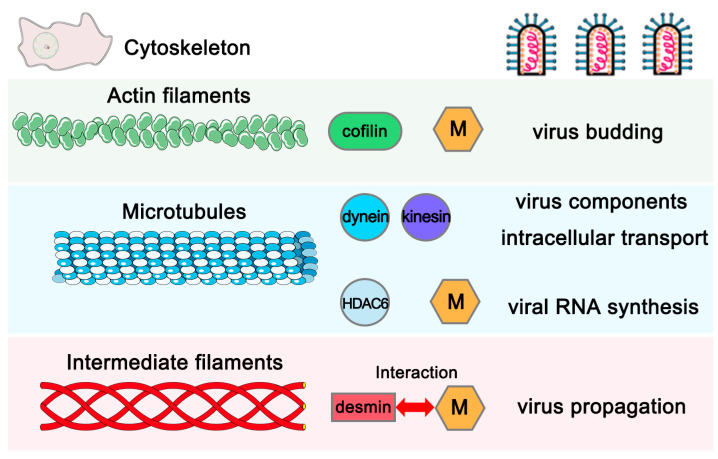
Graphical abstract illustrating that RABV-M exploits host proteins and cytoskeleton components to facilitate RABV infection. While the RABV utilizes cofilin and actin filament for efficient M-mediated virus budding, the M protein regulates HDAC6 expression and microtubule depolymerization to enhance viral RNA synthesis. Here, we report a novel physical and functional interplay between RABV-M and intermediate filament protein desmin that promotes RABV propagation.

As a neurotropic pathogen, RABV causes neuronal structural damage, neuronal dysfunction, and fatal encephalitis. After being bitten or scratched by an infected animal, RABV is transmitted from the wound site to the central nervous system (CNS) through the neuromuscular junction. It is thought that RABV can be transported in the infected neurons via either retrograde or anterograde trafficking. Once the virus reaches the CNS, it establishes a productive infection without interference by the peripheral immune system [1,26,27,28]. Over the past few decades, a series of host receptors of RABV have been identified, such as NCAM, p75NTR, and nAChR. Following attachment mediated via the glycoprotein (G), RABV is internalized into host cells via clathrin-mediated endocytosis, and the virus replication begins [1,18,28]. The cytoskeleton components, actin filaments, microtubules, and other related host proteins are targeted by RABV to facilitate the virus infection. First, the internalization and uptake of RABV requires an intact actin network, and the depolymerization of actin filaments inhibits the virus infection. Next, RABV utilizes the microtubules and the motor protein dynein/kinesin for intracellular transport [18,19,20,29,30]. During this stage, the M protein enhances the expression of cellular histone deacetylase 6 (HDAC6) to depolymerize the microtubules and facilitate viral RNA synthesis [31]. In addition, RABV induces the phosphorylation/inactivation of host cofilin to promote the polymerization of actin filaments, thus facilitating the M protein-mediated virus budding [32]. Here, we have identified the third member of the main cytoskeletal network, intermediate filaments (IFs), as also playing a role in RABV infection (Figure 6).

We found that the IFs component desmin is an interactor with the RABV-M protein that positively regulates the virus infection. It should be noted that the M protein from different RABV strains, including the attenuated strain RC-HL, standard strain CVS, and street strain GX-01 (Figure 1A), all interact with desmin, suggesting that desmin is a conserved target for the M protein of RABV strains, exhibiting different biological characteristics. Next, we showed that this physical interaction between RABV-M and desmin has an important functional role during the virus lifecycle. The overexpression of desmin in BHK-21 cells facilitated virus infection. Specifically, we observed a moderate increase in the mRNA expression levels of RABV N and M genes at 24 h post infection, however, the overexpression of desmin did not alter the intracellular proteins levels of RABV N and M. In comparison, the egress of progeny virions was significantly enhanced upon the addition of exogenous desmin at all three time points tested (Figure 2A). Correspondingly, the siRNA mediated suppression of endogenous desmin resulted in a significant reduction in the virus yields at all time points tested (Figure 3E). Taken together, these findings imply that desmin likely plays a role in the later stages of the virus lifecycle, such as virion assembly and budding. Indeed, we observed that endogenous desmin forms IF bundles throughout the cytoplasm of BHK-21 cells, and as expected, the RABV-M can distribute to the cell periphery and plasma membrane, in agreement with its role as the main driver of virus budding. Intriguingly, we found that a portion of the cytoplasmic RABV-M colocalizes on the desmin formed IF bundles (Figure 4). It is worth noting that RABV virion assembly begins with the M protein encapsulating the nucleocapsid core, followed by the M protein-mediated budding of the matured virions [1,5]. Thus, we hypothesize that the host IFs network, such as the other two cytoskeletal components, may also be usurped by the RABV M/desmin interaction, which facilitates the intracellular trafficking of RABV components.

The desmin-formed IFs are structural components of the sarcomeres in muscle cells and connect the intercellular junctions, such as the desmosomes, with the cytoskeleton [33,34,35]. Importantly, desmin plays an essential role in maintaining the structural and functional integrity of neuromuscular junction [36,37]. Since the neuromuscular junction is crucial for RABV transmission [1,26,27,28], it is tempting to speculate that IFs component desmin is beneficial to RABV transmission and pathogenesis. In support of this hypothesis, the in vivo experiments in mice indicated that the RABV challenge increases the expression level of the desmin mRNA and protein in the mouse brain. More importantly, the hippocampus is one of the cerebral regions that usually contains a high load of viral antigens [38,39], and our IHC analysis of the mouse hippocampus showed an obvious induction of desmin expression upon the RABV challenge.

IFs are involved in diverse cellular processes including vesicular transport, cell growth, cell migration, stress-related signaling pathways, and providing mechanical support to the cells. There are more than 70 genes encoding various IFs-proteins which can be classified into six types based on their structural, biochemical characteristics, and distribution [17,40,41,42]. Among them, type I IFs-protein keratin serve as a barrier in the epithelial tissues against microbial infection [43]; type V IFs-protein Lamin A/C are phosphorylated and reorganized by herpesvirus for the nuclear egress of viral nucleocapsid [44,45]; and type III IFs, which comprise desmin, vimentin, glial fibrillary acidic protein (GFAP), peripherin, and syncoilin, are particularly interesting due to their distinct distribution to specific cell types [17,42]. The vimentin-formed IFs (VIFs) have been linked to multiple stages of virus infection. For example, cell surface vimentin is involved in the invasion of SARS-CoV, SARS-CoV-2, PRRSV, DENV, and JEV; and the VIFs are required for the efficient replication and release of Enterovirus, FMDV, and SARS-CoV-2 [46,47,48]. In addition, it has been reported that desmin and vimentin are associated with the infection of TMEV, a neurotropic murine picornavirus. The TMEV virions are localized to the IFs, and the IFs network was rearranged to encompass the viral inclusion body during infection [49]. It is worth noting that type III IFs can be formed as either homopolymers or heteropolymers. While our current study reports that desmin is involved in the propagation of RABV, it is possible that other members in the type III IFs family also play roles in the RABV lifecycle, as well as desmin. Thus, the possible role and importance of the other type III IF proteins during the RABV infection remains to be addressed.

To sum up, our studies provide important and fundamental information regarding the impact of a RABV M/desmin interaction on the RABV lifecycle; however, the precise mechanism underlying the involvement of IFs during the RABV lifecycle still remains to be determined.

## Data Availability

The raw data of GST pull-down/MS have been deposited in the figshare database (https://doi.org/10.6084/m9.figshare.21937601.v1, accessed on 25 January 2023.). All the other relevant data are within the manuscript.
